# 3D Printing of Bioceramics for Bone Tissue Engineering

**DOI:** 10.3390/ma12203361

**Published:** 2019-10-15

**Authors:** Muhammad Jamshaid Zafar, Dongbin Zhu, Zhengyan Zhang

**Affiliations:** School of Mechanical Engineering, Hebei University of Technology, Tianjin 300130, China; jamshaid.zafer@yahoo.com

**Keywords:** bioceramics, additive manufacturing, scaffolds, bone tissue engineering

## Abstract

Bioceramics have frequent use in functional restoration of hard tissues to improve human well-being. Additive manufacturing (AM) also known as 3D printing is an innovative material processing technique extensively applied to produce bioceramic parts or scaffolds in a layered perspicacious manner. Moreover, the applications of additive manufacturing in bioceramics have the capability to reliably fabricate the commercialized scaffolds tailored for practical clinical applications, and the potential to survive in the new era of effective hard tissue fabrication. The similarity of the materials with human bone histomorphometry makes them conducive to use in hard tissue engineering scheme. The key objective of this manuscript is to explore the applications of bioceramics-based AM in bone tissue engineering. Furthermore, the article comprehensively and categorically summarizes some novel bioceramics based AM techniques for the restoration of bones. At prior stages of this article, different ceramics processing AM techniques have been categorized, subsequently, processing of frequently used materials for bone implants and complexities associated with these materials have been elaborated. At the end, some novel applications of bioceramics in orthopedic implants and some future directions are also highlighted to explore it further. This review article will help the new researchers to understand the basic mechanism and current challenges in neophyte techniques and the applications of bioceramics in the orthopedic prosthesis.

## 1. Introduction

Additive manufacturing or 3D printing has got attention in scaffold design and manufacturing for tissue engineering applications. Initially, this technique was developed by Sachs et al., to create the ink-jet freestyle printing towards the latter part of the 20th century [1]. Later on, it was extended in tailoring the perfect scaffolds on its user-friendly capabilities, which considered the transformation of computer aided design (CAD) information to a rapid and reliable production line of constructs with the coveted material, porosity, and measurements [2,3]. Moreover, it showed a time and cost-efficient potential coupled with interconnected structures, specifically hard tissue deformity regeneration.

Recently, clinical preliminaries and contextual analyses revealed its resounding accomplishments in the field of orthopedic bioengineering. While this procedure has shown significant potential, specific difficulties tend to enhance patient-particular scaffolds for standard acknowledgment in regenerative medicine [4,5,6].

During the past few decades, many advanced biomaterials were introduced in the biomedical field including different ceramic materials for the skeletal repair and reconstruction. These materials in the field of medical implants are often referred to as “bioceramics” [7]. Bioceramics are peculiar in nature due to their exceptional biological and osteoinduction properties. These materials are specific for scaffolds due to capability to create propagation, self-adhesion, distinction and bone tissues regeneration [8]. Furthermore, excellent chemical and mechanical properties such as better osteoconductivity, superior wear resistant and biocompatibility enabled them as a substitute for bone restoration, [9,10]. It can anticipate that bioceramics have a future due to increasing bone replacement operation per year due to increasing aging population [11].

The clinical importance of AM ceramic scaffold design and implantation envelops an invaluable method for quick and reliable production of hard tissue substitution replica of the biological context of natural bone [12]. In view of the way that customized scaffold can be prepared that suits an individual patient’s skeletal imperfection, layer-by-layer sintering is regarded as a lucrative discipline for the utilization of ceramic-based bone substitutes in regenerative medicine [13]. Besides, utilizing AM ceramic scaffolds as medication conveyance systems, it is becoming more attractive and relevant to the bioengineering environment [14,15,16].

This article is divided into six sections; Section 1 details the bioceramics potential, Section 2 offers an overview of the AM techniques used to fabricate ceramic parts. Section 3 presents achievements in the production of hydroxyapatite (HA); Section 4 depicts about tricalcium phosphate (TCP) and Section 5 describes about bioactive glass (BG) using different AM techniques. Section 6 concludes some important findings with some current challenges and future opportunities in this field.

## 2. Additive Manufacturing Technologies to Produce Ceramic Parts

**Figure 1:**
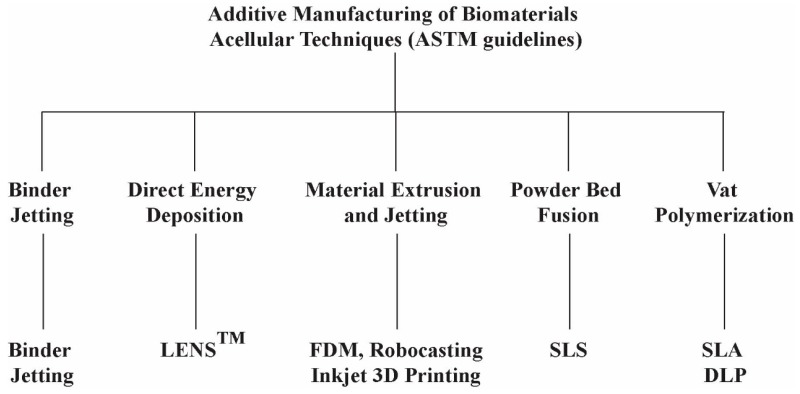
Different types of acellular techniques for biomaterials additive manufacturing (AM).

Additive manufacturing has been classified into two major classes such as acellular and cellular techniques for biomaterials. The cellular category includes the printing of live cells, while the acellular category does not consider any type of live cells in printing. Figure 1 shows different acellular AM techniques for biomaterials that have been classified as per recommendations of American Society for Testing of Materials (ASTM) [17]. The major AM techniques employed in the processing of bioceramics have been discussed in the following section.

### 2.1. Binder Jetting

**Figure 2:**
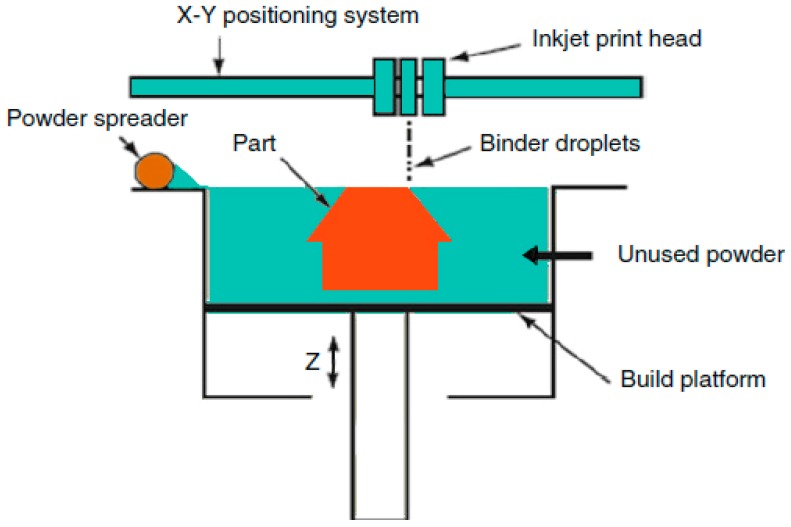
Schematic diagram of binder jetting mechanism [18].

Binder jetting technique was developed in the early 1990s at Massachusetts Institute of Technology (MIT) [18]. Figure 2 depicts the schematic of binder jetting. In this technique, the binder is selectively used from powder bed to create 3D objects. Binder jetting is a valuable technique for printing powder materials [19,20]. The particle size of the powder has a key influence on powder flowability in binder jetting.

For dry binder jetting, large size particles are preferred due to its outstanding flowability and less surface area. The powder size not only affects the flowability but also significantly affects the quality of the final product. Numerous researchers have reported less surface roughness using fine powder in the binder jetting. The effect of the powder shape is less, as compared to the powder size. However, spherical shape powders have better flowability and lesser friction compared to faceted powders [21,22,23].

### 2.2. Direct Energy Deposition (DED)

Direct energy deposition-based AM techniques uses energy into a small region to simultaneously deposit, melt and solidify the material such as wire or powder [24]. The direct energy source can be electrical, or laser beam can be used to melt the metal, ceramics or composite materials. Laser assisted Direct Deposition techniques such as laser cladding, laser engineered net shaping (LENS™), and laser melt injection are common examples of this technique.

**Figure 3:**
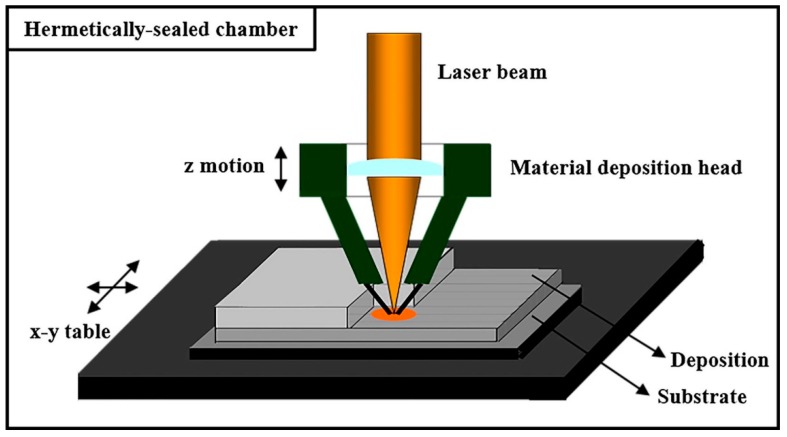
Typical Schematic representation of laser engineered net shaping (LENS) [26].

In ceramic Direct Energy Deposition (DED), the printing head of the apparatus contains a nozzle that feeds ceramic powder particles to the focal point of the laser beam. The ceramic powder melts and solidifies in layer-wise fashion on a substrate [25]. Figure 3 is the schematic illustration of LENS [26].

The major advantages of DED are better compatibility with a wide range of biomaterial viscosities, higher resolution and greater cell density that provide higher control of cell-to-cell adhesions [27]. Besides these advantages, DED has many challenges such as, low speed, cost, high complexity and limited capability to manufacture heterogeneous tissue parts [28]. Due to these challenges, the usage of DED is very limited as compared to other AM techniques particularly in bone tissue engineering. The DED technology needs more research to enhance its productivity.

### 2.3. Material Extrusion and Jetting

Extrusion assisted additive manufacturing deposits a continuous layer by layer deposition of ceramic loaded paste to create 3D objects. Various terms are used to refer to this technology for instance, Fused Deposition of Ceramics (FDC), Robocasting, Extrusion Freeform Fabrication (EFF), Direct Ink Writing (DIW), Slurry Deposition, and Dispense Plotting [29]. 

In Fused Deposition, dense ceramic particles (up to 60 vol%) are spread into a wax or thermoplastic filament after which the flexible filament is partly melted and extruded from a moving deposition head onto a fixed worktable layer-by-layer. However, in robocasting, ceramic slurry is ejected from a precise nozzle to form a filament that is directly deposited in a designed pattern to create complex 3D objects in a layer-by-layer fashion [30]. 

In another research work, an indirect Fused Deposition Modeling (FDM) method was applied to prepare ceramic parts. At the preliminary stage, FDM was used to prepare a honeycomb shaped polymer structural mold. Secondly, the ceramic slurry was permeated into the polymer mold-sintering to remove the mold. The porous ceramics made a correct pore size and porosity through this technique [31]. 

**Figure 4:**
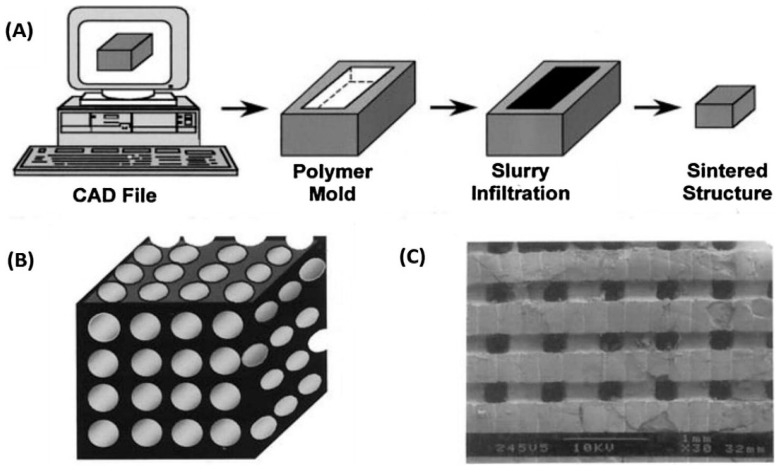
(**A**) Schematic of indirect Fused Deposition Modeling (FDM) processing of ceramic parts (**B**) Straight channels (**C**) Top view of sintered porous ceramic part [31].

Another technique named Extrusion-based bioprinting has also a greater potential in perspective of deposition and printing speed compared to other AM techniques. This technique is also beneficial to achieve better scalability in a shorter time [32], wide range flexibility of bioinks selection [33]. This is because developing new bioinks is a critical procedure for quick, sustainable and safe delivery of cells in a biomimetic microenvironment [32]. Besides many advantages, some complexities are also associated with this technology such as low resolution and shear stress effect on cells. The schematic of the process with part microstructure was shown in Figure 4.

**Figure 5:**
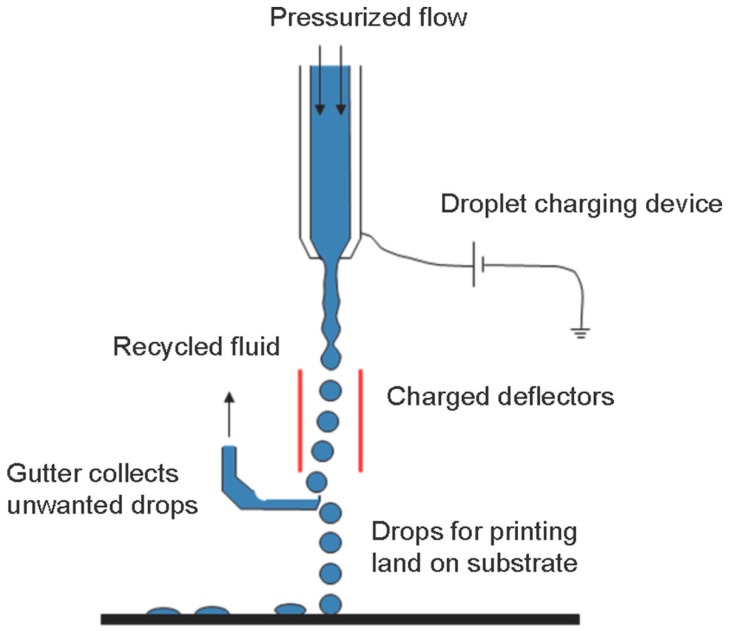
Schematic diagram depicts the basic working principle of ink-jet 3D printing [34].

The material jetting techniques are the “AM processes in which droplets of build material are selectively deposited” [17], that can be used to manufacture different kinds of ceramic parts. Inkjet 3D printing technology was among the first material jetting AM techniques that were employed for additive manufacturing of ceramic parts. It was developed by Sachs et al. in 1992 at MIT and defined as a process for the manufacturing of ceramic casting cores and shells using inkjet 3D printing [1]. Figure 5 shows the Schematic of ink-jet 3D printing [34].

### 2.4. Powder Bed Fusion

**Figure 6:**
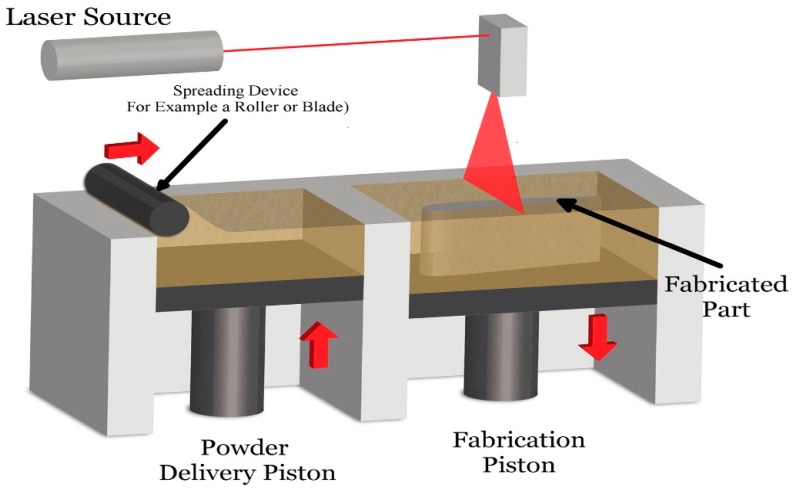
Schematic showing the working principle of powder bed fusion technique [40].

Powder bed fusion (PBF) technologies are among pioneer commercially used AM techniques created by the University of Texas USA. Selective laser sintering (SLS) based PBF technique [18], which melts the ceramic powder by laser energy source. The laser sintered the powder nearly to the melting point of the material to make each layer according to the given 3D design. The laser beam scans each new single layer of free-packed powder particles and consolidates them by sintering this process and proceeds in a layer-wise fashion to complete the final 3D object [35,36,37]. SLS is a powder bed fusion process have numerous applications in the bioengineering field such as to prepare customized products, biomedical implants as well as orthopedic implants [38]. The major disadvantage of SLS is the usage of higher temperatures that limits the insertion of biomaterial and cells into SLS scaffolds during the manufacturing process [39]. A schematic diagram illustrates the underlying operating system [40] of the powder bed fusion provided in Figure 6. While SLS technology is amended, the PBF method to increase machine efficiency.

### 2.5. Vat Polymerization (SLA)

Vat Polymerization also known as stereolithography (SLA) is a promising AM technique to fabricate tissue scaffolds in the field of regenerative medicine. The SLA technique has exceptional control over porosity of scaffolds, pore sizes, design flexibility, and interconnectivity [41]. Despite excellent advantages, numerous researchers have highlighted several challenges in scaffold manufacturing such as, difficult in creating micron-sized scaffold due to over curing and layer thickness. In addition, some of the frequently used biomaterials in bone tissue engineering have shown compatibility with SLA due to limitations in viscosity, refractive index and stability [42]. 

Some other problems such as some SLA processes light pixels restrict in-plane microstructure construction. Although indirect SLA have overcome this problem, it is a costly, time and material consuming process [43]. Li et al. have used indirect stereolithography to manufacture microporous β-TCP. The resin molds were prepared through this technique and filled with filled with aqueous thermosetting ceramic suspension for ceramic gel casting. The heat treatment process was used to remove the molds. Results have concluded that TCP scaffolds after sintering have shown desired porosity, shape and higher strength were obtained [44].

**Figure 7:**
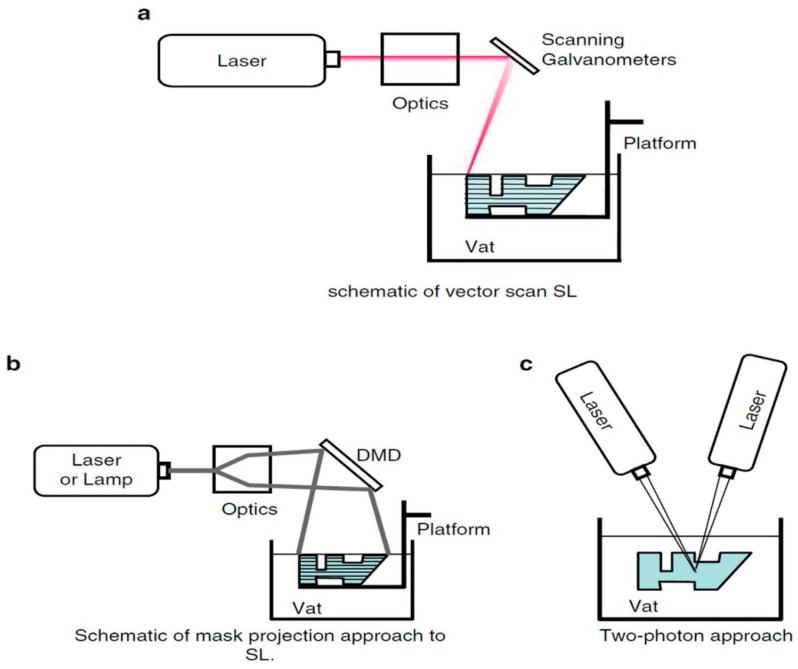
Schematic diagrams of three different techniques of photopolymerization [18].

Some other researchers have mentioned preparation of 3D objects by photo-curing a liquid resin through ultraviolet (UV) laser in a layerwise fashion [45,46]. The major advantage of this process includes better surface finish and accuracy [47]. A schematic of three different light sources used in stereolithography provided in Figure 7 [18]. Table 1 summarizing some basic bioprinting techniques for bone tissue engineering.

## 3. Additive Manufacturing of Bioceramics

In the last few decades, bioceramics have frequently been used in the restoration and replacements of injured tissues due to numerous advantages such as precise chemical composition, which has a vital role in the integration of hard and soft tissues [48,49]. Hydroxyapatite (HA) ${\mathrm{Ca}}_{10}$(${\mathrm{PO}}_{4}$)${\;}_{6}{\left(\mathrm{OH}\right)}_{2}$ is one bioceramic to have frequently been employed as a scaffold material for bone tissue engineering owing to its exceptional biocompatibility and resemblance to natural bone material [50,51,52]. It is often combined with a biopolymer or bioceramics to enhance binding interaction and mechanical properties of the material during the AM process [53,54].

Beta tri-calcium phosphate (β-TCP) is a suitable material for craniofacial defects owing to its excellent biodegradability, wear resistance and chemical bonding with the bone tissues under all load bearing conditions [55,56]. The critical challenge for β-TCP is to maintain the sintering temperature of 1100 °C. Above this temperature, beta tri-calcium phosphate (β-TCP) transforms to alpha tri-calcium phosphate (α-TCP) that is soluble and chemically unstable, as compared to β-TCP [57,58].

In addition, bioglasses are also extensively used in hard tissue implants due to their excellent bonding capability with hard and soft both tissues. Bioglasess are also extremely helpful in upregulating the osteogenesis, however, their application in load bearing bone defects are very limited due to their high brittleness, low fracture toughness and mechanical strength [59,60,61]. Properties of some frequently used ceramic material for bone tissue engineering illustrated in Table 2.

**Figure 8:**
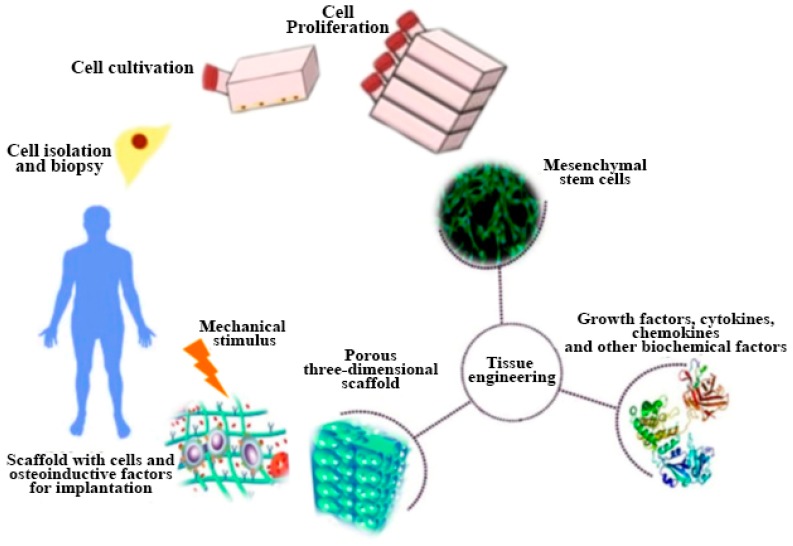
Schematic representation of bone tissue engineering [64].

The key factor affecting the performance of bioceramics is Ca to P ratio that affects the dissolution property. Calcium phosphates with lower Ca to P ratio (β-TCP) have higher solubility and acidic nature as compared to calcium-phosphate having high Ca to P ratio (HA) [62]. Table 3 shows that lower the Ca/P ratio higher the CaP dissolution [63]. Different bioceramics have been discussed in the following section such as hydroxyapatite, beta tri-calcium phosphate (β-TCP) and bioactive glass (BG) using different AM techniques. Figure 8 [64] shows complete process of bone tissue engineering.

### 3.1. Hydroxyapatite (HA)

Hydroxyapatite (HA) portrayed as ${\mathrm{Ca}}_{10}$(${\mathrm{PO}}_{4}$)${\;}_{6}{\left(\mathrm{OH}\right)}_{2}$, encompasses almost 65% of the entire bone mass. It is less toxic and more stable, as compared to other calcium-phosphate due to their desirable Ca to P ratio of 1.67. The hydroxyapatite has major inorganic part of human bone and teeth to develop the properties and novel applications of bioceramics for hard tissue replacements [65,66,67,68]. Numerous researchers have reported HA scaffolds in the bone and teeth transplants [69,70,71,72,73,74,75,76,77].

Laser Stereolithography has been identified as one of the most effective and frequently used AM techniques to produce complex HA parts. Barry et al. have prepared HA-based oligocarbonate dimethacrylate (OCM-2) composite scaffolds using helium-cadmium (HeCd) based laser technology. The outcomes referred the laser-based HA scaffolds provided fortified cell attachment inside the scaffold. Through laser machining, toxic leftovers were removed effectively through supercritical carbon dioxide (scCO_2_) to make scaffolds biocompatible. The HA based composite materials treated by scCO_2_ showed better attachment of cells in both vivo and vitro studies [78]. In a very recent study, a bio-ink was prepared for 3D printing by dispersing two different types of hydroxyapatites, nano hydroxyapatite (nHA) and deproteinized bovine bone (DBB) into collagen. Thereby, a porous structure was created by 3D printing. The chemical and physical properties of the materials, including biocompatibility and effect on the osteogenic differentiation of the human bone marrow-derived mesenchyme stem cells (hBMSCs) were investigated. Both nHA/CoL and DBB/CoL Bio-inks were used to print biomimic 3D scaffolds effectively. The outcomes from this study showed that the two types of hydroxyapatite composites which help hBMSCs proliferation and differentiation proved to be a promising candidate for a 3D scaffold bio-ink [79].

**Figure 9:**
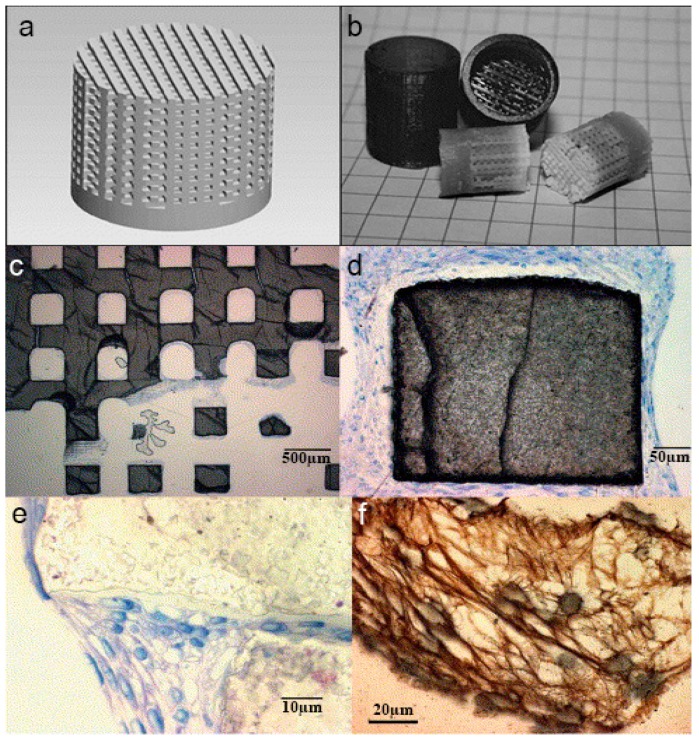
(**a**) Computer aided design (CAD) designed virtual structure of casting mold; (**b**) Resin casting molds manufactured by stereolithography and sintered hydroxyapatite (HA) structures; (**c**) SEM image of HA scaffold after culturing with MC3T3-E1 cells for 2 weeks, scaffold is visible in (dark grey) and cells (blue); (**d**) SEM image of strut of HA scaffold (grey), entirely attached with cells (blue/pink); (**e**) SEM image of microstructure of a crack between two struts, which was totally covered by MC3T3 cells (blue) and matrix created by the cells(pink); (**f**) SEM images of collagen produced by the cells (the microtome sectioning eliminates the mineral scaffold) [80].

Woeszn et al. fabricated microporous HA scaffolds having a pore size of 450 µm through stereolithography coupled with ceramic gelcating. A photosensitive liquid resin filled with water based thermosetting slurry was used in the mold. The mold resin and sintering were burnt to achieve the desired features. The final Scaffolds were seeded on MC3T3-E1 cells for 14 days under deep penetration of cells to achieve outstanding osteogenesis as shown in Figure 9 [80].

**Figure 10:**
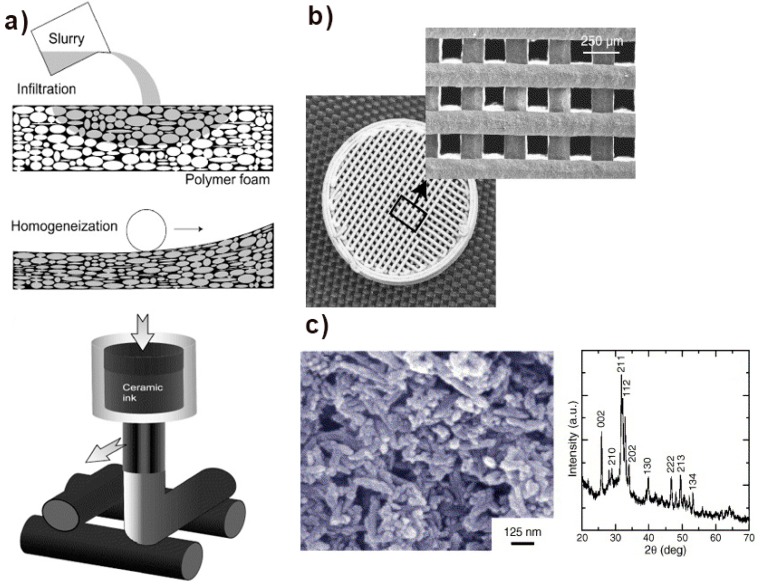
(**a**) Graphical representation of methods used in the manufacturing of porous ceramic scaffolds (**b**) Microstructure of HA scaffolds fabricated by robocasting (**c**) SEM micrograph and XRD of HA powders used in this process [81].

The AM based extrusion process is also very common to manufacture HA scaffolds. The robocasting based extrusion process contains ceramic ink in the form of water-based viscous slurry deposited on a robotic nozzle in layer-wise fashion based on computer-aided design. The process contains high loading of HA particles to minimize the cracks and distortion during sintering. Saiz et al. have fabricated HA scaffolds with controlled pore sizes through robocasting extrusion to find the optimum sintering temperature. The hydroxyapatite slurry was prepared by mixing 40–50 vol.% of HA powder in distilled water, 1.5 wt% of Darvan C dispersant, (∼7 mg/mL of solution) hydroxypropyl methylcellulose, an adequate amoqunt of polyethyleneimine (PEI) and at the end ${\mathrm{HNO}}_{3}$ or ${\mathrm{HN}}_{4}\mathrm{OH}$ to balance the pH level of the slurry. Results concluded that porous HA scaffolds manufactured with robocasting showed the sintering temperature should remain between 1100 °C∼1200 °C and no phase change was observed for firing 1300 °C for 3 h. The characteristics of printed scaffolds through this technique have been presented in Figure 10 [81].

Keriquel et al. have successfully printed the nano-HA scaffold in the mouse calvaria defect model in vivo using laser-assisted additive manufacturing. The printed cells showed the existence of vivacious blood vessels after bone defect treatment. The outcomes of this study demonstrated that laser-assisted bioprinting have perfectly treated bone defects. Through literature numerous authors have mentioned potential of this technology could offer new perspectives to additive manufacturing for the practical applications of bone tissue engineering [82].

### 3.2. Tricalcium Phosphate (TCP)

**Figure 11:**
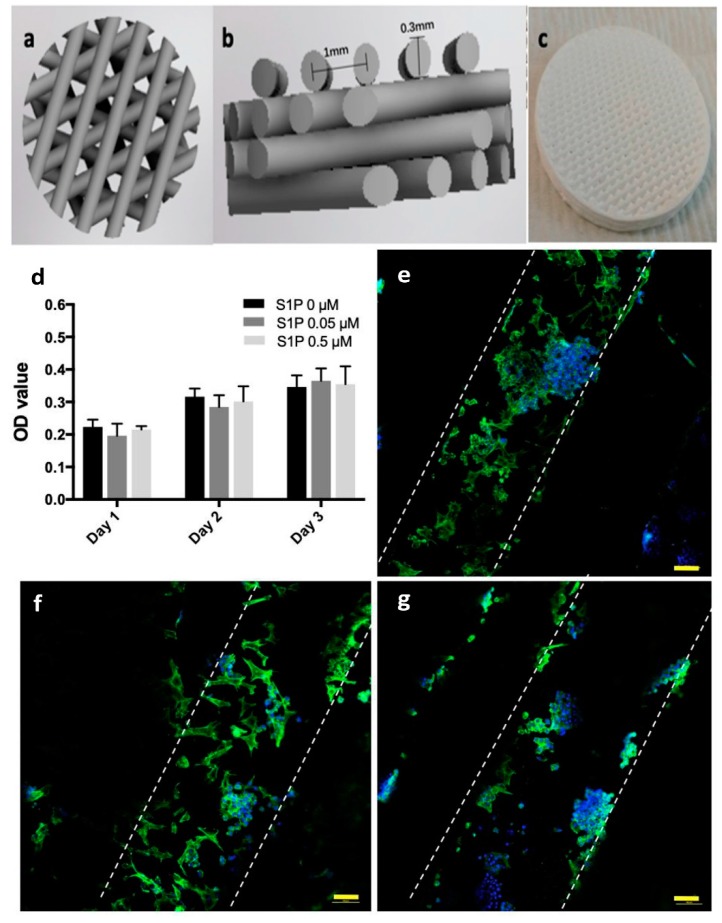
(**a**–**c**) Illustrates the schematic of 3D printed scaffold. (**d**) showing that there is no noticeable difference on viability of bone marrow-derived mesenchyme stem cells (BMSC) cells on additively manufactured scaffolds coated with S1P among the control group (S1P 0 mM) and other groups after 3 days. (**e**) S1P 0 mM group, (**f**) S1P 0.05 mM group, (**g**) S1P 0.5 mM group. Dyed blue area representing the cell nuclei and green area showing cytoskeletons. Edge of filaments showed by dotted lines [90].

Since the last two decades, beta tri-calcium phosphate (β-TCP) ceramic-based scaffolds have clinically accepted the bone graft replacement materials in several orthopedic and dental applications [83,84,85,86,87,88,89]. The TCP contains α, α′ and  β′ phases and Ca to P ratio is about 1.5. Cao et al. manufactured sphingosine 1-phosphate (S1P) coated β-TCP scaffold. Immunoregulation capability was tested on macrophages and rat bone marrow stromal cells of the coated scaffold was used to test osteogenic capability. The scaffold exhibit improved osteogenesis, better cell compatibility and also helpful to regulate the immune response as compared to traditionally manufactured scaffold. Figure 11 is the representation of 3D printed scaffold and its cell viability [90].

Bian et al. introduced a novel stereolithographic method to produce osteochondral beta-tricalcium phosphate/collagen scaffold. This bio-inspired scaffold manufactured by a combination of ceramic stereolithography (CSL) and gel casting using (β-TCP) and type-I collagen. Histological examination was performed to investigate the morphological properties between cartilage and bone. The obtained information from this examination were used to design biomimetic biphasic scaffolds. The pores size of β-TCP scaffolds varied between 700–900 µm with 50–65% porosity and compressive strength of 12 MPa. Physical locking formed by biomimetic transitional structure was used to achieve an adequate binding force among cartilage phase and a ceramic phase. The results concluded that CSL performed well in comparison with traditional techniques to get an ideal scaffold for bone tissue engineering applications [91]. 

**Figure 12:**
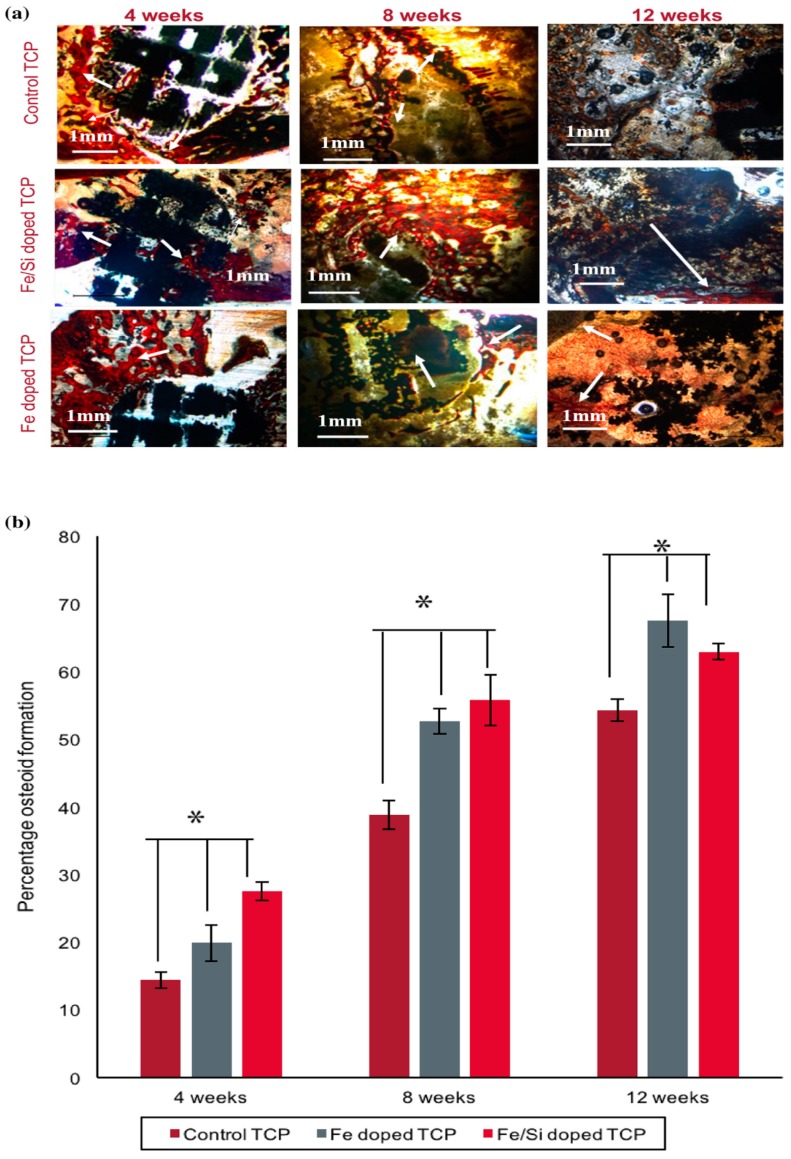
(**a**) Osteoid formation in pure and doped tricalcium phosphate (TCP) scaffolds after Modified Masson-Goldner trichrome staining after 4, 8, and 12 weeks. Black: prosthesis, orange and red: osteoid, bluish green: mineralized bone. Reddish-orange colors indicated by arrows showing new bone formation. Fe doping showed more bone mineralization as compared to others. (**b**) Histomorphic analysis showed Fe-Si doped TCP boosted initial stage osteoid formation for the period of 8 weeks and Fe doped TCP shows better mineralization of bone for 12 weeks of implantation [92].

In a recent study, Bose et al. have investigated the effect of ${\mathrm{Fe}}^{3+}$ and ${\mathrm{Si}}^{4+}$ dopants on the bio-mechanical properties of 3D printed β-TCP scaffold in a rat distal-femur for the period of 4, 8 and 12 weeks. The scaffold was fabricated by binder jetting technique using synthesized β-TCP powder. The outcomes from this analysis demonstrated that the addition of ${\mathrm{Fe}}^{3+}$ to TCP scaffold speed up the early stage bone restoration boosting type I collagen production. ${\mathrm{Si}}^{4+}$ doped TCP scaffold showed neovascularization after 12 weeks as shown in Figure 12. The finding from this study proved that ceramic powder-based scaffolds with improved chemistry has a promising future in bone defect restoration [92]. Tarafder et al. manufactured β-TCP scaffolds with 27%, 35% and 41% designed macroporosity with pore sizes of 500 µm, 75 µm and 1000 µm, respectively by 3D printing method. After that the scaffolds were sintered at the temperature of 1150 °C to 1200 °C in conventional and microwave furnaces to achieve mechanical strength. Microwave sintering heated scaffolds showed higher mechanical strength, as compared to conventional sintering. 

The pore size was examined by Human osteoblast cells. This study showed that a decline in pore size from 1000 to 750 and 500 µm has increased the cell density. Histomorphology tests in femoral defects of Sprague-Dawley rats revealed that the existence of both micro and macro pores accelerated the new bone construction. It was concluded that additive manufactured TCP scaffolds have outstanding potential in hard tissue engineering applications [93].

### 3.3. Bioactive Glass (BG)

Bioactive glasses (BG) are the type of bioceramics that exist in both nonporous and solid forms. The bio glass contains silicon dioxide, sodium oxide, calcium oxide and phosphorous. Different types of bioglass have been created by varying the vol.% of these components [94]. Silicate part plays have an essential role in the biocompatibility of bioactive glasses. The bio glass with 45–52 vol.% silicate has ideal bone-graft bonding [95]. The 45S5-bioglass is a well-known commercially available extensively used in bone replacement [96]. However, bioglasses have also some limitations due to poor mechanical properties and brittleness that makes them unsuitable for load-bearing applications, internally brittle and deficient mechanical strength. However, several researchers have reported different additives, such as metal, polymer and ceramic to enhance the mechanical properties [97,98,99,100,101].

Recently, Nommeots-Nomn et al. robocasted bioglass scaffolds with a 150 µm interconnected pore size (41–43% porosity) and measured compressive strengths were 32–48 MPa. The network connectivity (NC) of these scaffolds is like the 45S5 bioglass. In this process, ICIE16 and PSrBG compositions were used comprising < 50 mol% Si$O_{2}$ to maintain the amorphous structure and to achieve the required NC closer to 45S5 bioglass. The manufactured scaffolds were compared with 13–93 vol.% composition bioglass. The comparison highlighted that 3D porous scaffolds have similar NC values with 45S5 bioglass using two low silica contents. In addition, Pluronic F-127 binder could be accepted as a universal binder for bioactive glasses regardless of their composition and reactivity. Results also showed that ICIE16 and PSrBG based scaffolds are highly reactive and significantly enhanced the bone regeneration speed [102]. 

Padilla et al. used calcined bioglass suspension to fabricate porous scaffolds through integrating the stereolithography and gel-casting method. A polymeric negative mold was used via stereolithography to cast bioglass suspension with Darvan-811 (sodium polyacrylate) as a dispersant. The slurry containing 50 vol% was heated at 1100 °C for 55 s and later it was polymerized. The negative mold was removed by heat treatment. The scaffolds containing interconnected 3D channels of 400–470 µm length and 1.4 µm of pore diameter. The results illustrated that the entire interconnected porous scaffold was created by this method [103].

**Figure 13:**
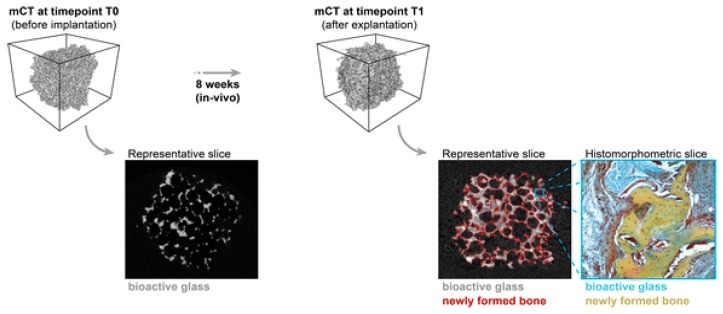
Histomorphometric and micro-CT analysis showing new bone development in polymer coated Bioglass (BG) scaffolds inserted in mice for eight weeks [104].

Westhauser et al. inspected the osteo-inductive properties of different polymer coated 3D-45S5 bioglass scaffolds. These scaffolds are seeded with human mesenchymal stem cells (hMSC) implanted into immunodeficient mice. The gelatin, cross-linked gelatin, and poly (3-hydroxybutyrate-co-3-hydroxyvalerate) type coatings were used. histomorphometry and micro-computed topography analysis were performed to evaluate the new formation after eight weeks of implantation. Although, every bioglass scaffolds showed noticeable bone regeneration. However, gelatin-coated bioglass scaffolds showed highest cell formation in comparison with other coated-bioglasses, as shown in Figure 13 [104]. Some latest researches on 3D printing of bioceramics have been compiled in Table 4.

## 4. Application of Bioceramics in Orthopedic Implants

Natural bone has self-repair capability after the damage. The smaller fractures heal itself correctly, however segmental bone defects (SBDs) lead to permanent paralysis [109,110]. SBDs fractures treated with autologous bone graft technique requires harvesting of non-vital bone, such as, the iliac crest. 

However, some complexities are also associated with bone grafting such as bone availability, the mismatch between harvested bone and affected site, morbidity of donor site results in poor integration [111]. Over the past three decades, a variety of synthetic materials have been introduced to overcome the complexities such as calcium phosphates (bioactive glasses) and hybrid bioceramics-polymer materials [112,113,114,115]. Table 5 showing different materials for bone tissue engineering.

**Figure 14:**
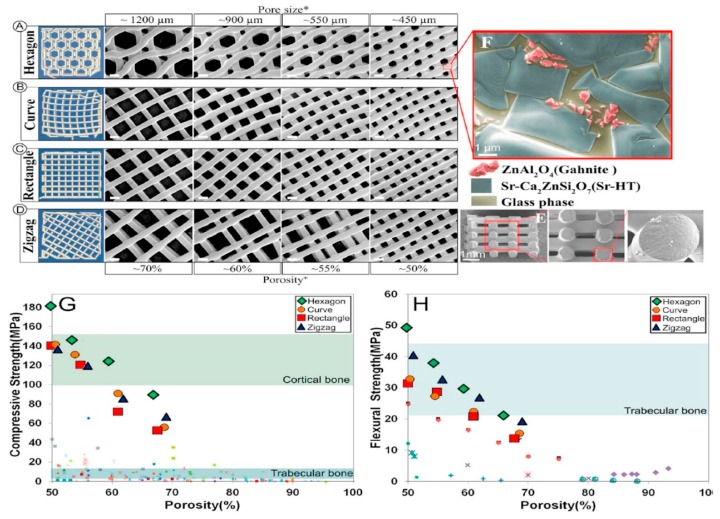
Models (left column) and SEM images of inspected scaffolds (scale bars: 500 µm unless stated otherwise); (**A**) Hexagonal; (**B**) curved; (**C**) rectangular and; (**D**) zigzag shape; (**E**) SEM images of fracture surface of a Sr-HT-Gahnite scaffold fabricated by robocasting; (**F**,**G**) the microstructure of Sr-HT-Gahnite scaffolds with distinct pore geometries vs porosity, and (**H**) flexural strength of Sr-HT-Gahnite scaffolds with hydroxyapatite and BG scaffolds [125].

Roohani-Esfahani et al. fabricated glass-ceramic scaffolds with hexagonal pore structure via extrusion-based AM method shown in Figure 14. The fabricated scaffolds have 150 times greater strength compared to polymeric-composite scaffolds and five times greater than ceramic-glass scaffolds having same porosity. The study has shown that these scaffolds have excellent capability to load-bearing and segmental bone defects treatment [125]. 

Fierz et al. prepared HA based cylindrical scaffolds ranging from nanometer to millimeter with straight channels and micro-pores through n-HA granules, ink-jet 3D printing AM technique. The structure of 3D-printed scaffolds is almost similar to human cortical and cancellous bone. The histological analysis has confirmed that osteogenic-stimulated progenitor-based 3D-printed scaffolds are suitable for clinical use [126]. 

**Figure 15:**
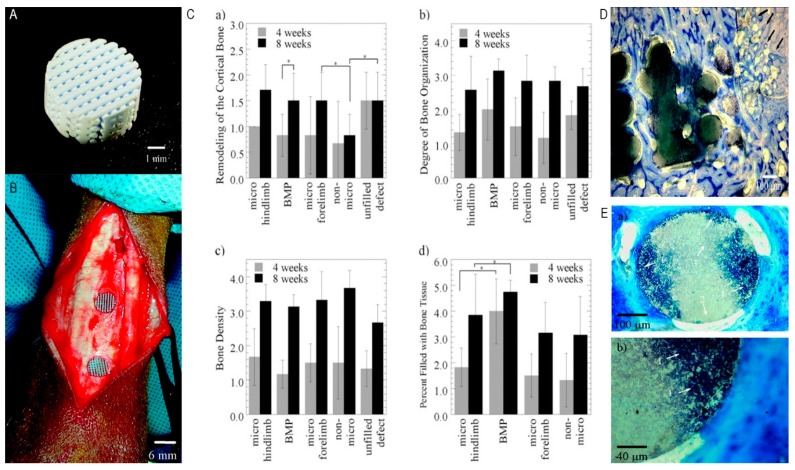
(**A**) Scaffold fabricated by directed deposition method. (**B**) The image of HA scaffold implantation in the metacarpal bone of goat. (**C**) BMP-2 and microporosity on cortical bone. (**D**) Image of BMP scaffold after 8 weeks representing the remodeling of the host bone, indicated by arrows. (**E-a**) and (**E-b**) are the images of histological section of micro hindlimb after 4 weeks indicating the staining of the microporous scaffolds at (**a**) low magnification and at (**b**) high magnification. Arrows indicate (1) stained and (2) unstained and (3) regions where staining extends into the scaffold [127].

In another study, a robocasting technique was utilized to transport bone morphogenic protein 2 (BMP-2). HA slurry and polymethylmethacrylate (PMMA) microspheres were mixed together to achieve controlled microporosity. Resins were eliminated by sintering the scaffolds at 1300 °C for 2 h. Thereafter, 10 µg of bone morphogenic protein 2 was added to the microporous scaffolds in goat bone for in vivo characterization for 4 and 8 weeks. Outcomes from this study showed great potential for manufacturing HA scaffolds containing interconnected porosity. Furthermore, the existence of bone morphogenic protein 2 and micro porosity upgraded scaffold osteogenesis ability as illustrated in Figure 15 [127].

**Figure 16:**
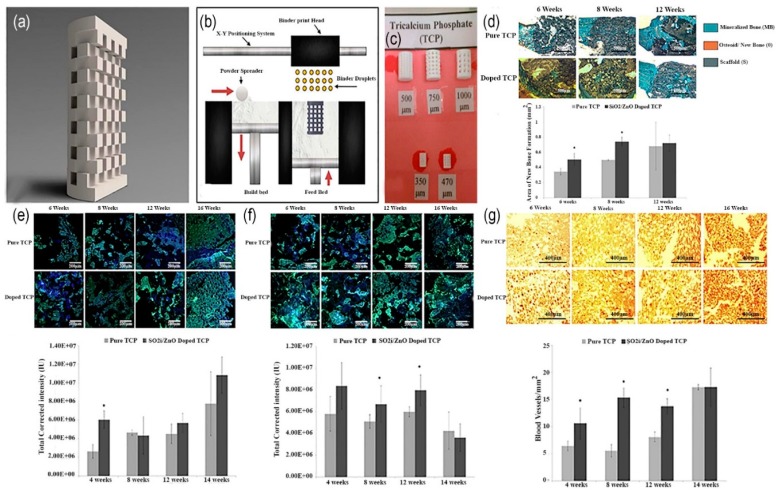
(**a**) CAD design used for 3D printing of porous scaffolds; (**b**) Schematic diagram illustration of 3D printing process (binder jetting); (**c**) Real 3D printed scaffolds, numbers indicating the pore size of scaffolds; (**d**) Staining of implant section via Goldner’s trichrome. Gray/brown color shows CaP implants, blue is mineralized implants and osteoid formation can be seen by orange color. Histomorphometry has done on trichrome sections (P < 0.1, where n = 3); (**e**) Confocal micrographs of collagen I formation (green) over the period of 16 weeks; (**f**) Confocal micrographs of osteocalcin (green). While blue color indicates counterstain for cell nuclei; (**g**) Light micrographs showing vWF staining (the dark red spots) [118].

Fielding et al. introduced (Si$O_{2}$/ZnO) doped three-dimensional composite TCP scaffolds with a pore size of 300 µm using binder jetting technique. The pure and (Si$O_{2}$/ZnO) doped 3D-printed TCP scaffolds implanted into a rat femur bone for the period of 6, 8 and 12 weeks to analyze the histomorphometry and Immunohistochemistry. Results have proved that combining Si$O_{2}$-ZnO dopants in TCP are best alternative to achieve osteoinductive properties of calcium phosphates (CaPs) for the clinical application of bone implants as shown in Figure 16 [118].

## 5. Challenges and Future Perspective

Despite all the achievements made in the past in 3D printing of tissue engineering, several challenges still exist. Challenges can be divided into two major categories: (1) 3D printing of biomaterials including live cells and (2) Post-implantation integration and functionality in vivo model. One of the most common problems during manufacturing is nozzle clogging in nozzle-based 3D printing techniques. To overcome nozzle clogging, printing precursor should have proper viscosity and need to be homogenous. Another problem is that the 3D printed constructs need to be adequately stable and mechanically stiff to ensure effective prosthesis. For instance, in hard tissue transplant, the scaffolds elastic modulus should be high enough to sustain its designed porosity and structure to help natural cell growth [128].

3D printed constructs for bone tissue engineering, being eventually implanted in a body, so these constructs also need to support vascularization to deliver sufficient amount of oxygen and nutrition to the cells in vivo to enhance the growth of newly implanted bone [129]. Vascularization plays a pivotal role in a successful bone tissue implant. However, it remains a daunting challenge in bone tissue engineering, particularly, in clinical application of large bone defects. Development of vascularized and clinically applicable bone substitutes with adequate blood supply, capable of inducing angiogenesis and sustaining implant viability remains a critical challenge. Since oxygen is only accessible to those cells through diffusion that are 100–200 µm from blood vessels, bioprinted constructs thicker than 400 µm face oxygenation problem. Therefore, it is a critical task to provide ideal conditions to help vascularization in implanted bone constructs. There is a need for some extensive research to completely understand the mechanism of the biological system of bone. Thus, manufacturing a biomimetic vascularized bone that mimics the native bone can be helpful to overcome these hurdles. Due to the ability of bioprinters utilizing several print-heads loaded with different cell types, introducing vasculature was made possible to a 3D printed construct [130,131,132].

Recently, nozzle-based 3D-printers enabled the printing of endothelial cells using multiple bioinks for the development of thick vascularized [133,134]. Especially, digital light processing (DLP) based 3D bioprinting can offer extraordinary speed, scalability and resolution for printing complex 3D structures with micrometer resolution [135,136]. For instance, Zhu et al. printed well-designed vascular channels without using perfusion or sacrificial materials by utilizing a rapid microscale continuous optical bioprinter (μCOB). In this method, glycidyl methacrylate-hyaluronic acid (GM-HA) and GelMA-cell laden bioinks were used to create channels and channel adjacent regions. From the outcomes of this study, researchers were able to demonstrate the progressive formation of endothelial network and formation of the lumen-like structures in vivo/vitro model. Anastomosis between the bioprinted endothelial network and circulation was observed with functional blood vessels featuring red blood cells [137]. Moreover, hypoxia is also having an important role in vascularization and bone regeneration process. Hypoxia belongs to the family of Hypoxia-Inducible Transcription Factors (HIFs) [138]. Kuss et al. utilized short-term hypoxic conditions to endorse vascularization in a hybrid 3D printed scaffold of polycaprolactone/hydroxyapatite (PCL/HAp) and stromal vascular fraction (SVF) derived cell laden bioink [139].

Another type of challenge is regulatory hurdles, customized 3D printing technology entails series of difficulties in the regulatory approval field. Though, it is urgent for the managing authorities to establish appropriate laws and regulations to ensure sustainable progress of 3D printing technology. At present, 3D-printed scaffolds and tissues are used for evaluation and screening purposes in animal models.

## 6. Conclusions

In summary, this review outlined the latest researches on development of 3D printing of bioceramics for bone tissue engineering, current state of the art is also discussed. Extensive amount of research on 3D bioprinting over the past 10 years highlighted its wide range of applications and potentials in bone tissue engineering. Although, plethora of goals have been accomplished in 3D printing of bioceramics, but it is still in its emerging stage. 

However, to deal with challenges such as vascularization, and printing related problems, further research on development of bioinks, integration of different 3D bioprinting technologies, improvement of the mechanical properties of existing bioceramics, development of composites with excellent biocompatibility and better understanding of bonding mechanism between bone mineral and collagen are some primary areas of concern that can help to improve the applications of 3D printing in bone tissue engineering.

Recently, a very limited number of bio-printed products have been commercially available. Due to the rapid expansion of this industry in the last few years, it is foreseeable that more bio-printed constructs will ultimately become commercially available to help wide range of patients suffering from different kind of diseases. The technical problems related to clinical requirements and materials selection are mentioned above, multidisciplinary research will be required to tackle those problems and to comprehensively understand the potential of bioprinting in bone tissue engineering.

## Figures and Tables

**Table 1:** Summary of major 3D-bioprinting techniques for bone tissue engineering.

Technique	Principle	Advantages	Drawbacks
Inkjet	A liquid binding material is selectively deposited in layer-wise fashion into the powder bed to create three dimensional objects.	Ability to print biomaterials with low viscosity, high resolution, fast manufacturing speed, low cast	Intrinsic inability to deliver a continuous flow, low cell densities, lack of functionality for vertical objects
Extrusion	This process involves extruding the material in viscous form to create 3D objects	Capability to print variety of biomaterials, Capable of printing high cell densities	Applicable to viscous liquids only
Laser-assisted	In this technique, a laser beam stimulates a specified area of target to fabricate 3D objects	High resolution, capable of printing both solid and liquid phase biomaterials	High cost, low speed, high complexity, thermal damage due laser irritation
Stereolithography (SLA)	In this method an ultraviolet (UV) laser beam selectively hardens the photo-polymer resin to construct 3D models in layer-wise fashion	Nozzle free method, high cell viability, high accuracy, Printing time independent of complexity, high cell viability, high accuracy	UV light can cause toxicity to cells, during photo curing damage to cells, Applicable to photopolymers only

**Table 2:** A brief review of ceramic materials and its properties used in 3D printing of Scaffolds.

Materials	Precursors	Properties
Hydroxyapatite (HA)	Poly (acrylic acid), photo-curable resin, polycaprolactone, poly (lactic acid) etc.	Higher biocompatibility, differentiation and proliferation, better cell adhesion
Tricalcium Phosphate (TCP)	Hydroxypropyl methylcellulose, polyethylenimine, polymethacrylate, etc.	In physiological environment better biocompatibility and degradation, lower compressive strength
Bioactive glasses alkali-free bioactive glass, 45S5 BG,13-93 bioactive glass, 6P53B glass	Polycaprolactone, methylcellulose, poly (lactic acid)	Improved bioactivity in vitro and in vivo for the bone tissue growth

**Table 3:** Characteristics of main CaPs used as bone substitutes and cements [63].

Name	Formula	Ca/P Ratio	Water Solubility at 25 °C, g/L
Monocalcium Phosphate			
Monohydrate (MCPM)	Ca(H_2_PO_4_)_2_, H_2_O	0.50	∼18
Anhydrous (MCPA)	Ca(H_2_PO_4_)_2_		∼17
Dicalcium phosphate			
Dihydrate (DCPD)	CaHPO_4_, H_2_O	1.00	∼0.088
Anhydrous (DCPA)	CaHP_4_		∼0.048
Tricalcium Phosphate			
Alpha α-TCP	(α)${\mathrm{Ca}}_{3}$(PO_4_)_2_	1.50	∼0.0025
Beta β-TCP	(β) ${\mathrm{Ca}}_{3}$(PO_4_)_2_		∼0.0005
Hydroxyapatite (HA)	Ca_5_(PO_4_)_3_OH	1.67	∼0.0003

**Table 4:** Overview of 3D printed bioceramics for bone tissue engineering.

Material	Process	In Vivo/In Vitro Model	Key Findings	Ref.
HA + liquid sodium polyacrylate + photopolymer	A ball crusher was used to milled all the materials for 12 h to make a slurry with solid content of 10~60 wt%. The ceramic scaffold was fabricated by using digital light processing (DLP) technique	Mouse osteoblast precursor cells (MC3T3.E1) were cultured in the condition of α-MEM (10% fetal bovine serum 4% penicillin-streptomycin)	3D printed scaffold showed better biocompatibility, adhesion, differentiation and also able to promote osteoblast proliferation	[105]
Biphasic calcium phosphate (HA/β-TCP = 60:40) + HMPC+ polyethylenimine + Zr$O_{2}$	Extruded at pressure of 600 kPa with printing speed of 100 mm/min. Constructs were sintered at 1100 °C	Investigated on osteoblast like sarcoma cells for cytotoxicity and for differentiation potential of the scaffolds hMSCs cells were used	Better mechanical properties of scaffolds at 10% (*w*/*w*) of Zr$O_{2}$ was observed with improved BMP-2 expression.	[106]
β-TCP/polycaprolactone	β-TCP powder with 550 nm particle size were used to fabricate 350 µm pore size cylindrical scaffolds.	Composite scaffolds were tested using human fetal osteoblast cells (hFOB) for 3, 7 and 11 days of incubation period	Enhanced early bone formation and effective for controlled alendronate release	[107]
β-TCP/sphingosine 1-phosphate (SIP)	The scaffolds were printed in four layers and in different sizes to fit in 6-well and 12-well plates. Printed scaffolds were sintered at 1100 °C for 3 h.	Immunoregulation capability was investigated on macrophages and the osteogenic capability was tested on rat bone marrow stromal cells of the coated scaffolds.	Good biocompatibility, improved bone regeneration process	[90]
Bioactive glass/alginate	Composite scaffolds of type 13-93 bioactive glass (13-93 BG) and sodium alginate (SA) were prepared with mass ratio of 0:4, 1:4, 2:4 and 4:4 under mild conditions for bone regeneration.	The apatite mineralization abilities of the 13-93 BG/SA scaffolds were tested by soaking scaffolds in simulated body fluid (SBF), using 200 mL $g^{-1}$ of scaffold mass, at 37 °C for 0 and 10 days.	Improved porosity and reduced shrinkage ratios	[108]
Bioglass (BG)/gelatin/cross linked-gelatin/ploy (3-hydroxybutyrate-co-3-hydroxyvalerate)	Three different types of 3D-polymer coated BG (45S5-type) scaffolds were fabricated by the well-established foam replica method and coated with the biopolymers.	Osteo-inductive properties of 3D-45S5 bioglass scaffolds were investigated by seeding human mesenchymal stem cells (hMSC) implanted into immunodeficient mice for the period of 8 weeks.	Under standard conditions biopolymer coated 3D 45S5 BG scaffolds have ability to induce bone formation. Gelation coated scaffolds showed the best results.	[104]

**Table 5:** Additive manufacturing (AM) materials for bone prostheses.

Material	Binder	Layer Thickness	References
TCP	Aqueous based	20 µm	[93]
HA	-	100 µm	[116]
α/β-TCP modified with 5 wt% hydroxypropymethylcellulose	Water	100 µm	[117]
β-TCP, Si$O_{2}$-ZnO-dope β-TCP	Water based binder	20 µm (β-TCP) 30 µm (SiO_2_-ZnO-doped β-TCP)	[118]
HA	α-n-butyl cyanoacrylate (NBCA)	-	[119]
TCP	20% (*v*/*v*) phosphoric acid	125 µm	[120]
TTCP/β-TCP	25% citric acid	100 µm	[121]
α-TCP	10 wt.% phosphoric acid	50 µm	[122]
HA/Maltrodextrin	Water based	175 µm	[123]
HA & Maltrodextrin/apatite-wollastonite glass	Water based	100 µm	[124]
